# Enhancing Care for Older Adults Through the Medicare Annual Wellness Visit

**DOI:** 10.1093/geroni/igaf122.1065

**Published:** 2025-12-31

**Authors:** Jennifer Wolff, Aleksandra Wec, Nicole Werner

## Abstract

The Medicare Annual Wellness Visit (AWV) is a preventive care service which collects patient-reported information and supports the creation of a personalized prevention plan to address identified patient needs. Recent studies indicate that having a billed AWV is associated with increases in preventive care services such as vaccinations, cancer screening, and dementia diagnosis. However, there is variation in patient subpopulations who receive the AWV and the opportunities to enhance implementation of the visit to better meet the needs of older adults. This session offers insights into the AWV from different contexts, including population-, health system-, and implementation-level. The first session uses a nationally representative sample of Medicare beneficiaries to provide an overview of the differential uptake of the AWV across Medicare insurance coverage type (Traditional Medicare vs. Medicare Advantage), highlighting heterogenous impact by age, race, and health status. The next session examines the AWV at a health system level, by drawing on electronic health record data to identify the frequency of follow-up actions taken after memory concerns are reported during the AWV. The third session presents a pilot study seeking to improve dementia care pathways by testing workflow enhancements via nurse-led AWVs. Attendees of this symposium will take away key insights for potential improvements to the AWV. The session discussant will offer thoughts on how the AWV can meet the needs of a diverse population of older adults while aligning with health system priorities.

